# Prevalence, Factor Structure and Correlates of DSM-5-TR Criteria for Prolonged Grief Disorder

**DOI:** 10.3389/fpsyt.2022.880380

**Published:** 2022-05-18

**Authors:** Julia Treml, Elmar Brähler, Anette Kersting

**Affiliations:** ^1^Department of Psychosomatic Medicine and Psychotherapy, Medical Faculty, University of Leipzig, Leipzig, Germany; ^2^Department of Psychosomatic Medicine and Psychotherapy, University Medical Center of the Johannes Gutenberg University Mainz, Mainz, Germany; ^3^Department of Psychiatry and Psychotherapy, Medical Faculty, University of Leipzig, Leipzig, Germany

**Keywords:** prolonged grief disorder (PGD), prevalence, DSM-5-TR, bereavement, grief/loss

## Abstract

**Background:**

Prolonged Grief Disorder (PGD) is now included in Section II of the *Diagnostic and Statistical Manual of Mental Disorders*, 5th Edition, Text Revision (DSM-5-TR). To understand the health burden and then allocate economic and professional resources, it is necessary to provide epidemiological data for this new disorder. This is especially relevant since the new diagnostic criteria differ from its predecessors, which may affect the generalizability of previous findings. More information on the characteristics of people suffering from PGD is also beneficial to better identify individuals at risk. This study, therefore, aimed to estimate the prevalence of the new PGD criteria in a representative population-based sample, evaluate the factor structure, sociodemographic, and loss-related correlates of PGD caseness and explore possible predictors.

**Methods:**

Out of a representative sample of the German general population (*N* = 2,531), *n* = 1,371 (54.2%) reported to have experienced a significant loss throughout lifetime. Participants provided sociodemographic data and loss-related characteristics. PGD symptoms were measured using items from the German versions of the Prolonged Grief Scale (PG-13) and the Inventory of Complicated Grief (ICG), which could be matched to the DSM-5-TR criteria for PGD.

**Results:**

The conditional prevalence of PGD was 3.4% (*n* = 47). The most frequently reported symptoms were intense emotional pain and intense yearning or longing for the deceased. The confirmatory factor analysis confirmed a unidimensional model of PGD. Regression analysis demonstrated that time since the death, the relationship to the deceased, and unpreparedness for the death were significant predictors of PGD.

**Conclusion:**

Although the prevalence of 3.4% using the new diagnostic criteria is lower than the prevalence previously suggested by a meta-analysis, PGD remains a substantial disorder in the general population. In particular, the loss of a partner or child increases the risk for PGD, as does unpreparedness for the death of a loved one. Clinicians should pay particular attention to these high-risk groups. Further clinical implications are discussed.

## Introduction

A substantial body of research has been directed toward the investigation of Prolonged Grief Disorder (PGD), which is characterized by intense longing or yearning for the deceased or preoccupation with the deceased, coupled with intense emotional pain causing significant impairment in functioning beyond 12 months after the loss (1). For many years, scientists have called for the inclusion of PGD into the classification systems such as the Diagnostic and Statistical Manual of Mental Disorders (DSM) while proposing different criteria sets [e.g., (2–4)]. The DSM-5 workgroup on Trauma/Stress-Related and Dissociative Disorders first decided that more research was necessary on specific diagnostic criteria for a bereavement related disorder and introduced the term *Persistent Complex Bereavement Disorder* (PCBD), which was included in Section III “Conditions for Further Study” within the DSM-5. As such, PCBD was not a formal disorder but created to encourage research into the condition (5–7). Based on the collected evidence from this research, the American Psychiatric Association (APA) finally approved the inclusion of PGD in Section II in the DSM-5-TR in November 2020, thereby replacing the criteria for PCBD in Section III (1, 8). The DSM-5-TR was just released in March 2022 (9) and the present article focuses on its new PGD criteria.

To understand the health burden and then allocate economic and professional resources, it is necessary to provide epidemiological data for this new disorder. A previous meta-analysis estimated the prevalence of PGD after natural losses to be about 10% (10). Investigating only people bereaved by unnatural losses such as suicide, homicide, or accidents, led to a substantially higher rate of 49% (11). However, the included studies showed a high risk of external validity bias, a large degree of between-study heterogeneity, and all estimates were based on earlier definitions and criteria sets of PGD, which are now outdated. Therefore, previous prevalence estimates might be less reliable. Furthermore, prior studies were mostly based on convenience samples, specific subgroups or clinical samples leading to a wide variability in the conditional prevalence of PGD. To obtain a more representative result, more population-based studies investigating the current PGD criteria are needed, such as the recent study by Rosner et al. (12). They found a conditional prevalence of PGD of 3.3%.

The introduction of the new PGD diagnoses within the DSM-5-TR also necessitates an investigation of the dimensionality of the new criteria. The first exploratory investigation of PGD indicated a unidimensional construct (8), which was recently supported by a study comparing a one-factor model to a two-factor-model (13). The authors hypothesized that the one-factor model would yield the best statistical fit, which was confirmed in their study. This unidimensional model needs to be replicated in further confirmatory factor analyses.

Additionally, more information on the characteristics of people suffering from PGD is beneficial, to better identify individuals at risk. Previous research revealed inconsistencies regarding factors associated with PGD. Some studies indicated for instance that older bereaved are more vulnerable than younger [e.g., (14, 15)], while others found no association with *age* (16) or even contrary results suggesting that younger bereaved are more vulnerable (17). Other sociodemographic variables investigated include *gender, years of education, employment status*, or *monthly income*. The results are likewise ambiguous with some studies suggesting that female gender, being less educated or having a low income is positively associated with PGD [e.g., (14, 15, 18–21)], while other results indicated no association [e.g., (16, 18, 22–24)].

The same inconsistencies can be found the loss-related variable *cause of death*. Some study results suggest that violent deaths lead to an increased risk for PGD (13, 23, 25), while again others found no association [e.g., (16, 26, 27)]. The cause itself might not be the determining factor but the *perception of the death experience* and *preparedness* for death. Researchers typically categorize deaths as violent or non-violent, depending on how the death occurred, which may not necessarily be consistent with the perception of the bereaved. For instance, a natural loss due to an illness that is accompanied by suffering and pain may be perceived as violent but would be categorized as non-violent (28). A first examination found no association between the perception of the death experience and PGD. However, the sample was small and a reinvestigation of the perception of the death experience as a factor in a larger sample might be beneficial. Studies investigating perceived preparedness for the death of a loved one suggest that preparedness might be a protective factor (29). In line with this finding, study results demonstrated that people who had lost a family member in subjectively unexpected circumstances showed a greater severity of PGD than those who had expected the death of a family member (30). This is consistent with recent study results that showed that the unexpectedness of a death was significant predictor of PGD caseness (31). Another study also found unexpected losses to be significantly related to greater PGD symptom severity. However, the association was weak and further studies are needed (32). Interestingly, in all of these studies, about half of the participants reported that the death of their loved one was unexpected, including those losses due to a disease which might be a further indicator that the subjective perception of the death is a better predictor than the objective cause of death.

A well-studied loss-related variable is the personal *relationship to the deceased.* Most findings indicate that losing a child or partner is positively associated with PGD compared to the loss of other relatives (e.g., parents, siblings) or friends (14, 15, 21, 33, 34). Another loss-related variable is *time since loss*, for which some studies found no association (24, 26, 35), whilst others found an association with PGD (15, 16, 23, 27). For instance, Newson et al. found more time since loss to be associated with PGD (15), whereas Schaal et al. found less time since loss to be associated with more grief (23). The inconsistencies among previous findings could be due to small sample sizes or the use of convenience samples, specific subgroups (e.g., military samples, students, widowers/widows, and elderly) or clinical samples. It is thus important to conduct population-based studies including a large sample with various bereaved subpopulations among all age groups and both genders.

The current study therefore aimed to (a) estimate the prevalence of PGD according to the new DSM-5-TR criteria in a population-based sample representative for the general population of Germany, (b) evaluate the assumed unidimensional model of PGD, and (c) explore sociodemographic and loss-related correlates of PGD caseness and investigate possible predictors.

## Materials and Methods

### Participants and Procedures

Data of a representative sample of the population in Germany was collected between November 2017 and February 2018 with the assistance of a demographic consulting company (USUMA, Berlin, Germany). The three-stage random sampling procedure involved: (1) random selection of 258 regional sample point areas representing the different regions of the country, (2) random selection of target households within sample point areas based on a random-route procedure, (3) random selection of one member within target households fulfilling the inclusion criteria (age ≥ 14, able to read and understand the German language) based on a Kish-selection-grid. The Kish-selection-grid technique aims to sample individuals on the doorstep. The sample was constructed to be representative in terms of gender, age, and education.

A total of 5,093 target persons were approached by one of 223 trained interviewers. If not at home, a maximum of three further attempts were made to contact the selected person. Potential participants received oral and written information about the study and provided written informed consent. For target persons under the age of 18, additional parental informed consent was acquired. Sociodemographic information was collected in a face-to-face interview. Thereafter, participants filled out self-report questionnaires, and interviewers assisted in case of questions. The study and the procedures were approved by the local ethical review board (Leipzig University, Medical Faculty; AZ: 418/17-ek, 23.10.2017) and conducted in accordance with the declaration of Helsinki.

A total of 2,531 (49.7%) people between the ages of 14 and 93 years agreed to participate. Reasons for non-response were: households could not be reached (*n* = 731, 14.4%), households declined participation (*n* = 840, 16.5%), (c) target persons could not be reached (*n* = 181, 3.6%), (d) target persons declined participation (*n* = 804, 15.8%). Six interviews were not applicable for analyses. Of the 2,531 participants, 1,371 (54.2%) reported having experienced the loss of a close person (e.g., a partner, relative or good friend). Table 1 provides an overview of the participant characteristics. Bereaved individuals were mostly middle-aged, and 59% were women. The great majority (55.2) was employed and about 80% indicated less than 12 years of education. The majority reported to have lost a parent (45.5%) or grandparent (15.8%, grouped as “others”), and natural non-violent deaths were reported most frequently (83.4%) as the cause of death.

**TABLE 1:** Participant characteristics and differences between people with and without probable PGD.

Bereaved sample						

	Total	No PGD	Probable PGD	Test	*p*-value	Effect size
	(*N* = 1,371)	(*N* = 1,324)	(*N* = 47)			
Gender				χ^2^(1) = 7.77	**0.005**	**ϕ = 0.077**
Male, *n* (%)	562 (41.0)	552 (41.7)	10 (21.3)			
Female, *n* (%)	809 (59.0)	772 (58.3)	37 (78.7)			
Age, M (*SD*)	54.56 (17.28)	54.23 (17.26)	63.94 (15.30)	*t*(1,306) = -3.89	**<0.001**	**d = 0.572**
Education				χ^2^(1) = 2.80	0.095	ϕ = −0.046
<12 years, *n* (%)^a^	1,087 (79.6)	1,045 (79.2)	42 (89.4)			
≥12 years, *n* (%)^b^	279 (20.4)	274 (20.8)	5 (10.6)			
Employment status				χ^2^(2) = 11.06	**0.004**	**ϕ = 0.093**
Employed, *n* (%)^c^	751 (55.2)	709 (56.6)	15 (31.9)			
Unemployed, *n* (%)	94 (6.9)	85 (6.8)	5 (10.6)			
Retired, *n* (%)	515 (37.9)	458 (36.6)	27 (57.4)			
Monthly household net income				χ^2^(2) = 2.03	0.363	ϕ = 0.040
<1,250 EUR, *n* (%)	214 (16.3)	204 (16.1)	10 (21.7)			
1,250–2,500 EUR, *n* (%)	588 (44.7)	566 (44.6)	22 (47.8)			
≥2,500 EUR, *n* (%)	512 (39.0)	498 (39.3)	14 (30.4)			
Time since death in months, M (*SD*)	128.1 (138.1)	130 (139.0)	74.5 (97.3)	*t*(53.45) = 3.85	**<0.001**	**d = 0.401**
Deceased is				χ^2^(2) = 113.79	**<0.001**	**ϕ = 0.298**
Partner, n (%)	204 (15.2)	179 (13.8)	25 (53.2)			
Child, *n* (%)	32 (2.4)	24 (1.9)	8 (17.0)			
Other, *n* (%)^d^	1,104 (82.4)	1,090 (84.3)	14 (29.8)			
Cause of death				χ^2^(1) = 0.34	0.558	ϕ = 0.016
Natural, non-violent, *n* (%)	1,138 (83.4)	1,100 (83.5)	38 (80.9)			
Unnatural, violent, *n* (%)	226 (16.6)	217 (16.5)	9 (19.1)			
Perception of the death^e^						
Violent, M (*SD*)	3.39 (2.06)	3.33 (2.04)	4.15 (2.39)	*t*(48.55) = −2.33	**0.024**	***d* = 0.398**
Suffering, M (*SD*)	3.55 (1.97)	3.51 (1.94)	4.15 (2.41)	*t*(48.28) = −1.81	0.077	*d* = 0.324
Drawn-out, M (*SD*)	3.23 (2.00)	3.21 (1.98)	3.36 (2.37)	*t*(48.44) = −0.44	0.661	*d* = 0.075
Unprepared, M (*SD*)	4.82 (2.01)	4.76 (2.01)	5.63 (1.79)	*t*(1,297) = −2.88	**0.004**	***d* = 0.433**

*Percentages were calculated from valid cases.*

*^a^Equals primary/secondary school.*

*^b^Equals college/university.*

*^c^Including students.*

*^d^Parent, sibling, grandparent, other relative, friend.*

*^e^Higher scores indicate perception of a more difficult death and being less prepared, range 1–7.*

*All significant variables are represented in bold.*

### Measures

Sociodemographic data included gender, age, education, employment status, and income. Bereaved participants were also asked to provide data on the characteristics of the deceased and the loss (e.g., relationship to the deceased, cause of death, time since death) using a self-constructed questionnaire. The cause of death was categorized as violent if participants indicated losing someone to suicide, accident, or homicide. If participants lost more than one significant person, they were asked to refer to the person whose loss affected them the most. There was no maximum time since loss criterion (e.g., no more than 10 years) to include all participants who have lost a close loved one.

Grief symptoms were assessed using items from the German versions of the Prolonged Grief Scale (PG-13) (3, 36) and the Inventory of Complicated Grief (ICG) (37, 38). Items could be matched to the DSM-5-TR criteria for PGD (see Table 2 for corresponding items). To meet the DSM-5-TR criteria for PGD, at least one of the two Criterion B items, at least three of the eight Criterion C symptoms, and the Criterion D item should be endorsed for those who experienced the death of a loved one at least 12 month prior (Criterion A) (1, 8). All Criterion symptoms were tapped by one of the included items, except for one symptom [C4 criterion: “Intense emotional pain (e.g., anger, bitterness, sorrow) related to the death],” which was captured by two items. The highest score on one of these two items was used to represent the C4 criterion. All items were rated on 5-point scales (1 = not at all to 5 = several times a day/overwhelmingly). The items of the ICG were recoded to a scale from 1 to 5 (instead of 0–4).

**TABLE 2:** Frequency of occurrence of single symptoms of PGD.

			Symptom present					
DSM-5-TR			Population-based (*N* = 2,531)		Bereaved (*N* = 1,371)	*M*	*SD*	Factor loading
		
	Criteria for PGD	Matched item	*N*	%	%			
A	The death of a person close to the bereaved *at least 12 months* previously.		1,250	49.4	91.2			
B	Since the death, there has been a grief response characterized by *one or both* of the following, to a clinically significant degree, nearly every day or more often for at least the last month:		227	9.0	16.6			
	1. Intense yearning/longing for the deceased person	In the last month, how often have you felt yourself longing or yearning for the person you lost?^a^	217	8.6	15.8	2.31	1.15	0.601
	2. Preoccupation with thoughts or memories of the deceased person	Have you been so preoccupied with the person who has died that it was difficult for you to do the things you normally do?^b^	48	1.9	3.5	1.41	0.80	0.779
C	As a result of the death, *at least 3* of the following 8 symptoms have been experienced to a clinically significant degree since the death, including nearly every day or more often for at least the last month:		309	12.2	22.5			
	1. Identity disruption (e.g., feeling as though part of oneself has died	Do you feel confused about your role in life or feel like you don’t know who you are anymore (i.e., feeling like that a part of you has died)?^a^	90	3.6	6.6	1.73	1.00	0.786
	2. Marked sense of disbelief about the death	In the past month, how often have you felt stunned, shocked, or dazed by your loss?^a^	96	3.8	7.0	1.65	1.00	0.712
	3. Avoidance of reminders that the person is dead	In the past month, how often have you tried to avoid reminders that the person you lost is gone?^a^	124	4.9	9.0	1.83	1.10	0.588
	4. Intense emotional pain (e.g., anger, bitterness, sorrow) related to the death^c^	In the past month, how often have you had intense feelings of emotional pain, sorrow, or pangs of grief related to the lost person?/Do you feel bitter over your loss?^a^	298	11.8	21.7	2.48	1.27	0.703
	5. Difficulty with reintegration into life after the death (e.g., problems engaging with friends, pursuing interests, planning for the future)	Do you feel that moving on (e.g., making new friends, pursuing new interests) would be difficult for you now?^a^	90	3.6	6.6	1.6	0.97	0.819
	6. Emotional numbness (i.e., absence or marked reduction in the intensity of emotion, feeling stunned) as a result of the death	Do you feel emotionally numb since your loss?^a^	90	3.6	6.6	1.68	0.99	0.875
	7. Feeling that life is meaningless as a result of the death	Do you feel that life is unfulfilling, empty, or meaningless since your loss?^a^	91	3.6	6.6	1.70	1.01	0.888
	8. Intense loneliness (i.e., feeling alone or detached from others) as a result of the death	Do you feel alone or lonely without the deceased?^b^	160	6.3	11.7	1.99	1.19	0.789
D	The disturbance causes clinically significant distress or impairment in social, occupational, or other important areas of functioning.	Have you experienced a significant reduction in social, occupational, or other important areas of functioning (e.g., domestic responsibilities)?^a^	121	4.8	8.8			
Prevalence according to the diagnostic algorithm			47	1.9	3.4			

*^a^PG-13 item.*

*^b^ICG item.*

*^c^Criterion C4 was captured by two items, the highest score is used to represent C4.*

The participants’ perception of their loved one’s death and their preparedness for the death were measured with the Perception of Circumstances Surrounding the Death (PCSD) Scale (28). The scale assesses on a 7-point Likert scale whether the death was perceived as peaceful or violent (1 = peaceful, 4 = moderate, 7 = violent), whether the loved one suffered (1 = minimally, 4 = moderately, 7 = extremely), and whether the dying process seemed drawn-out (1 = over very quickly, 4 = moderate, 7 = extremely prolonged). The last item assesses whether the bereaved felt prepared for the death (1 = well prepared, 4 = somewhat prepared, 7 = totally unprepared). Higher scores indicate the perception of a more difficult death and more unpreparedness.

### Statistical Analyses

All statistical analyses were conducted using the Statistical Package for Social Sciences, version 25 (IBM^®^ SPSS^®^), including the software Analysis of Moment Structures, version 25 (IBM^®^ SPSS^®^ Amos). The significance level was set to α = 0.05.

To estimate the prevalence of PGD, the number of participants fulfilling the respective criteria were counted. A symptom was considered present if scores were ≥ 4 (at least “quite a bit/at least once a day) (3). Each symptom was dichotomously coded as “not present” (0) or “present” (1). For exploratory reasons, percentages of endorsement and means of each item were also calculated.

To evaluate the dimensionality of the PGD criteria, a confirmatory factor analysis (CFA) was conducted based on structure equation modeling using the software AMOS, version 25. We predicted a one-factor model representing a unidimensional construct. Because the χ^2^-test as a global measure for the model fit is highly influenced by sample size, the following close fit indices and corresponding cut-off criteria were used to evaluate the model fit: Comparative Fit Index (CFI) and Tucker-Lewis Index (TLI), with values > 0.90 indicating acceptable and ≥ 0.95 indicating excellent model fit; Root Mean Square Error of Approximation (RMSEA) including the 90% confidence interval, with values between 0.05 and 0.08 indicating acceptable and ≤ 0.05 indicating excellent model fit, and Standardized Root Mean Square Residual (SRMR), with values < 0.10 indicating acceptable and < 0.05 indicating excellent model fit (39, 40). As the original model assuming uncorrelated errors indicated only partially acceptable fit to the data, minor model modifications were necessary. Thus, the total sample was split into two random samples using the SPSS 25 random selection procedure. Model modifications were performed in the first split-half sample. The model modifications were guided by a review of modification indices and only considered if they were theoretically plausible. Afterward, the final model was cross-validated in the second split-half sample.

To examine whether PGD caseness differed in socio-demographic (e.g., gender, education) or loss-related variables (e.g., cause of death, relationship to the deceased), χ^2^-test or *t*-tests were used and effect sizes were calculated. Φ-values between 0.1 and 0.2 can be considered small effects, between 0.3 and 0.4 medium effects and ≥ 0.5 large effects. Whereas *d*-values from 0.2 to 0.4 can be considered small effects, between 0.5 and 0.7 moderate effects, and ≥ 0.8 large effects.

Subsequently, a binary logistic regression analysis was performed with PGD caseness as the dependent variable and factors with significant relationships identified by the abovementioned analysis as predictor variables.

## Results

The conditional prevalence of PGD among all bereaved using the DSM-5-TR diagnostic algorithm was 3.4% (*n* = 47), within the population-based sample (including non-bereaved) 1.9%. Frequency distributions showed that intense emotional pain (e.g., anger, bitterness, sorrow) related to the death, intense yearning/longing for the deceased person and intense loneliness (i.e., feeling alone or detached from others) as a result of the death were frequently endorsed items (21.7–8.6%). The least endorsed item was preoccupation with thoughts or memories of the deceased person (1.9%; see Table 2).

To evaluate the facture structure, a CFA was performed for cases with complete data, resulting in a total sample of *N* = 1,333. A one-factor model assuming uncorrelated errors indicated poor model fit (CFI = 0.891, TLI = 0.859, RMSEA = 0.152, 90% CI: 0.145, 0.160, SRMR = 0.057, χ^2^ = 1116.294, *p* < 0.001). Thus, minor model modifications were performed in the first split-half sample (*n* = 655). The final model, allowing for correlations of unique variances between the items B1, C2, C3, and C4, showed an acceptable fit to the data in the first split-half sample [χ^2^(29) = 131.017, *p* < 0.001; CFI = 0.980; TLI = 0.969; RMSEA = 0.073, 90% CI: 0.061, 0.086; SRMR = 0.024]. These correlations were considered theoretically plausible as they differed from all other items in terms of similarities of the wording (“in the past months, how often have you …”) and the response scale, which may reflect a non-random measurement error. These four items are all answered on a scale from 1 = not at all to 5 = several times a day, whereas all others are answered from 1 = not at all to 5 = overwhelmingly. The cross-validation in the second split-half sample (*N* = 678) confirmed the model fit (χ^2^(29) = 147.800, *p* < 0.001; CFI = 0.975; TLI = 0.962; RMSEA = 0.078, 90% CI: 0.066, 0.090; SRMR = 0.030). The χ^2^-test was not considered as a global measure of model fit, as it is strongly influenced by sample size.

Regarding differences in sociodemographic and loss-related variables between participants with and without probable PGD, we found that PGD was more prevalent among women [χ^2^(1) = 7.77, *p* = 0.005], older participants [*t*(1,306) = −3.89, *p* < 0.001] and those more recently bereaved [*t*(53.45) = 3.85, *p* < 0.001] (see Table 1). Caseness also varied by employment status [χ^2^(2) = 11.06, *p* = 0.004] and the relationship to the deceased [χ^2^(2) = 113.79, *p* < 0.001]. People with probable PGD also indicated perceiving the death as more violent [*t*(48.55) = −2.33, *p* = 0.024] and being less prepared for the death compared to those without PGD [*t*(1,297) = −2.88, *p* = 0.004].

Binary logistic regression analysis demonstrated that time since the death, the relationship to the deceased, and unpreparedness for the death were significant predictors of PGD (Table 3). More time since the loss lowered the risk for PGD [odds ratio (OR) = 0.994, 95% confidence interval (CI) = 0.990–0.998]. Those bereaved by a partner or child were more likely to experience PGD than those who lost another loved one (e.g., parent, sibling, friend) (OR = 8.094, 95% CI = 3.314–19.770, OR = 14.110, 95% CI = 4.581–43.460, respectively). Furthermore, being unprepared for the death of the loved one significantly predicts PGD (OR = 1.223; 95% CI = 1.012–1.479). Taken together, these variables explain 26.5% of the variance.

**TABLE 3:** Binary logistic regression analysis of predictive variables for PGD.

	*B*	SE	Wald	df	*p*	Exp (B)	95% CI	
							Lower	Upper
Gender	0.647	0.392	2.719	1	0.099	1.910	0.885	4.112
Age	0.019	0.017	1.215	1	0.270	1.019	0.985	1.054
Time since death in months	−0.006	0.002	8.185	1	**0.004**	0.994	0.990	0.998
**Employment status is (vs. employed)**								
Unemployed	0.282	0.544	0.269	1	0.604	1.326	0.456	3.854
Retired	0.990	0.633	2.447	1	0.118	2.693	0.778	9.313
**Deceased is (vs. other)**								
Partner	2.091	0.456	21.061	1	**<0.001**	8.094	3.314	19.770
Child	2.647	0.577	21.269	1	**<0.001**	14.110	4.581	43.460
**Perception of the death**								
Violent	0.119	0.079	2.277	1	0.131	1.126	0.965	1.314
Unprepared	0.202	0.097	4.352	1	**0.037**	1.223	1.012	1.479

*SE, standard error; df, degree of freedom; CI, confidence interval. All significant variables are represented in bold.*

## Discussion

One aim of this study was to explore the prevalence rate of the new DSM-5-TR criteria for PGD in a population-based sample to provide more epidemiological data. Previous prevalence estimates are predominantly based on old criteria sets. Differences between the criteria sets may threaten generalizability of previous epidemiological research to the current DSM-5-TR criteria, making studies examining the new PGD criteria indispensable. Additionally, given the wide variability of the conditional prevalence of PGD in prior studies, mainly with specific subgroups, clinical samples and/or community based samples, this study aimed to obtain a more representative result by using a population-based sample. Another aim was to investigate the dimensionality of the new disorder and examine correlates and possible predictors of PGD caseness.

The first main finding was a conditional prevalence of PGD of 3.4% (*n* = 47), which is lower than the prevalence of 9.8% found by Lundorff and colleagues in their meta-analysis (10). Population-based studies are less likely to attract people with severe mental health problems than convenience samples. Therefore, a lower prevalence is possible [e.g., (41–43)]. However, the rates within the meta-analysis were all based on various previous criteria sets of PGD, most of the studies used cut-offs for diagnosing PGD instead of a diagnostic algorithm and only four out of 14 included studies used a population-based approach to ensure representativeness (10).

In a recent validation of the new DSM-5-TR criteria for PGD by Prigerson et al. (8), prevalence rates of 4.4% (similar to ours; Yale Bereavement Study), 10.9% (Oxford Grief Study), and even 15.3% (Utrecht Bereavement Study) were found. The first two prevalence rates were found in community-based samples. The last prevalence rate of 15.3% was found in a sample recruited *via* mental health professionals, in which higher prevalence rates are expected. Boelen and Lenferink (21) also investigated the prevalence of the new PGD criteria and found a prevalence of 10.1%. Study participants were recruited *via* announcements on the Internet as well as bereavement care workers, which could have led to a higher prevalence. Population-based studies can help minimize such a selection bias and provide a more representative estimate for the general population. Moreover, our result aligns with the prevalence of 3.3% (*n* = 30) found in another population-based study (12). This indicates that PGD is a substantial disorder in the general population and our findings add to few epidemiological data available for the new PGD criteria.

The most frequently reported symptom in our study was intense emotional pain related to the death (C4, 21.7%), followed by intense yearning or longing for the deceased (B1, 15.8%). Preoccupation with thoughts or memories of the deceased person (B2) was the least endorsed item and only reported by 3.5% of the bereaved. This was also the case within the Yale Bereavement Study, raising the question of whether this item should be one of the core items of criterion B (8) or whether only intense yearning or longing for the deceased should be the key criterion.

Our second aim was to investigate the factor structure of PGD. To the best of our knowledge, this is the first study is to test a one-factor model in a large representative sample. As expected, the one-factor model was confirmed in the CFA, indicating a unidimensional construct. This result confirms the findings of an exploratory factor analysis by Prigerson et al. (8), as well as recent findings by Lenferink and colleagues (13). This result is also in accordance with studies examining the PG-13 and the translated versions (of which most items were used in this study) that also found a one-factor model [for an overview see (44)].

Regarding the last aim, we first explored differences between participants meeting the PGD criteria with those not meeting the criteria. The preliminary analyses indicated that PGD was more prevalent in women, older people and more recently bereaved. Caseness also varied by employment status and the relationship to the deceased. These findings are in line with previous findings using former criteria for PGD [e.g., (14, 15)]. Contrary to some previous studies [e.g., (23, 25)], we found no significant difference for the cause of death (violent/non-violent), but for the perception of the death as violent. People meeting the criteria of PGD reported perceiving the death as more violent than those not meeting the criteria did. This indicates that the categorization of deaths as violent or non-violent according to the cause of death may not necessarily be consistent with the perception of the bereaved. We argue that the latter might be a more adequate predictor of PGD. However, since the number of participants meeting the PGD criteria was relatively small, the statistical power might have been too low to detect possible effects of the cause of death.

In a final step, we entered these variables into a regression analysis. The results revealed that age and gender were not significant predictors when controlling for other factors such as relationship to the deceased. An explanation might be that the relationship to the deceased is the crucial variable. In our study, losing a partner increased the risk for PGD by 8.1 times compared to losing another close person, and the loss of a child increased the risk by as much as 14.1 times. Similar results were obtained in another population-based study (31). The probability of losing one’s partner increases with age, with women losing their partners more frequently than the other way around, as they have a longer life expectancy (45). In addition, the probability of losing a child (who might already be an adult) also increases with age. This could contribute to the age and gender differences found within the χ^2^- and *t*-tests. That these variables are no more predictive of PGD when controlling for relationship to the deceased might indicate that age and gender do not have an inherent influence on the development of PGD. Furthermore, the results showed that more time since the loss seems to lower the risk for PGD slightly, but the odds ratio was close to one, indicating a small and possibly irrelevant effect. This could indicate that once PGD is present, it remains stable over time. This is in line with findings by Prigerson et al. (8) who concluded that PGD is unlikely to remit over time. The variable time since the loss showed a great variability within our study which enabled the evaluation of the predictive power across a considerable time period.

Regarding the perceptions of the circumstances surrounding the death, only unpreparedness remained a significant predictor of PGD. Our results showed that being unprepared increased the relative probability of PGD by 22%. This aligns with previous studies that indicated that high levels of perceived preparedness for the death of a loved one might be a protective factor for better post-loss adjustment (29), whereas unpreparedness for or subjective unexpectedness of a death increases the risk for PGD (29–32). In case of terminally ill patients, health care providers can support family members and caregivers by facilitating the participation in the patients’ care and providing clear information on the impending death, as these actions have been found to promote preparedness for death (46). This could help reduce the risk of developing PGD in people bereaved by illnesses (e.g., cancer).

As mentioned above, since the number of participants meeting the PGD criteria was relatively small, the statistical power to detect further predictors might have been too low. Future research should therefore reassess these variables in a larger group of people suffering from PGD. Further methodological limitations include that our results are based on self-reported questionnaires rather than clinician-administered structured interviews. The exclusive use of self-report measures could have evoked a bias due to misinterpretation of questions. Furthermore, the retrospective assessment of the loss-related variables may be affected by recall bias. Moreover, the cross-sectional study design eliminates any causal conclusions, calling for longitudinal studies. And lastly, psychiatric comorbidities and general mental health problems were not assessed. Therefore, the extent to which possible comorbid psychopathology influenced the results is an unknown, challenging interpretation of the results.

Notwithstanding these considerations, our findings extend and confirm previous investigations. The study’s major strength is the population-based setting, with a sample constructed to be representative in terms of gender, age, and education. Most studies on PGD include predominately female participants leading to an underrepresentation of men. Our results demonstrate that PGD represents a substantial disorder among the bereaved, even though the prevalence rate of 3.4% using the new diagnostic criteria is lower than the prevalence rate of 9.8% found in a meta-analysis based on previous criteria sets (10). Since PGD is a rather new disorder, it might be relatively unknown to health care providers compared to other mental disorders such as depression or anxiety. However, this substantial disorder should not be neglected. Intense emotional pain related to the death, intense yearning/longing for the deceased person, and intense loneliness were the most prevalent symptoms in our study. In particular, people who lost a partner or child are at high risk of developing PGD, as are those who were unprepared for death. Clinicians should pay particular attention to these high-risk groups and refer them to treatment if needed. Short instruments for PGD such as the PG-13-R (8) could be easily implemented in primary care to identify those in need.

## Data Availability Statement

The raw data supporting the conclusions of this article will be made available by the authors, without undue reservation.

## Ethics Statement

The studies involving human participants were reviewed and approved by the Local Ethical Review Board (Leipzig University, Medical Faculty; AZ: 418/17-ek, 23.10.2017). Written informed consent to participate in this study was provided by the participants’ legal guardian/next of kin.

## Author Contributions

JT and AK contributed to conception and design of the study. EB organized the database. JT performed the statistical analysis and wrote the first draft of the manuscript. AK supervised the study. All authors contributed to manuscript revision, read, and approved the submitted version.

## Conflict of Interest

The authors declare that the research was conducted in the absence of any commercial or financial relationships that could be construed as a potential conflict of interest.

## Publisher’s Note

All claims expressed in this article are solely those of the authors and do not necessarily represent those of their affiliated organizations, or those of the publisher, the editors and the reviewers. Any product that may be evaluated in this article, or claim that may be made by its manufacturer, is not guaranteed or endorsed by the publisher.
